# A Nanomule Peptide Carrier Delivers siRNA Across the Intact Blood-Brain Barrier to Attenuate Ischemic Stroke

**DOI:** 10.3389/fmolb.2021.611367

**Published:** 2021-03-26

**Authors:** Brett A. Eyford, Chaahat S. B. Singh, Thomas Abraham, Lonna Munro, Kyung Bok Choi, Tracy Hill, Rhonda Hildebrandt, Ian Welch, Timothy Z. Vitalis, Reinhard Gabathuler, Jacob A. Gordon, Hans Adomat, Emma S.T. Guns, Chieh-Ju Lu, Cheryl G. Pfeifer, Mei Mei Tian, Wilfred A. Jefferies

**Affiliations:** ^1^ Michael Smith Laboratories, University of British Columbia, Vancouver, BC, Canada; ^2^ The Vancouver Prostate Centre, Vancouver General Hospital, Vancouver, BC, Canada; ^3^ Centre for Blood Research, University of British Columbia, Vancouver, BC, Canada; ^4^ The Djavad Mowafaghian Centre for Brain Health, University of British Columbia, Vancouver, BC, Canada; ^5^ Department of Medical Genetics, University of British Columbia, Vancouver, BC, Canada; ^6^ Department of Neural and Behavioral Sciences and Microscopy Imaging Core Lab, Pennsylvania State College of Medicine, Hershey, PA, United States; ^7^ Centre for Comparative Medicine, University of British Columbia, Vancouver, BC, Canada; ^8^ Department of Chemistry, University of British Columbia, Vancouver, BC, Canada; ^9^ Bioasis Technologies Inc., Guilford, CT, United States; ^10^ King’s College London, London, United Kingdom; ^11^ Department of Urologic Sciences, University of British Columbia, Vancouver, BC, Canada; ^12^ Department of Microbiology and Immunology, University of British Columbia, Vancouver, BC, Canada; ^13^ Department of Zoology, University of British Columbia, Vancouver, BC, Canada

**Keywords:** stroke, peptide-oligonucleotide conjugate, MTfp, blood-brain barrier, NOX4, siRNA

## Abstract

The blood-brain barrier (BBB) hinders the distribution of therapeutics intended for treatment of neuroinflammation (NI) of the central nervous system. A twelve-amino acid peptide that transcytoses the BBB, termed MTfp, was chemically conjugated to siRNA to create a novel peptide-oligonucleotide conjugate (POC), directed to downregulate NOX4, a gene thought responsible for oxidative stress in ischemic stroke. The MTfp-NOX4 POC has the ability to cross the intact BBB and knockdown NOX4 expression in the brain. Following induction of ischemic stroke, animals pretreated with the POC exhibited significantly smaller infarcts; accompanied by increased protection against neurological deterioration and improved recovery. The data demonstrates that the MTfp can act as a nanomule to facilitate BBB transcytosis of siRNAs; where the NOX-4 specific siRNA moiety can elicit effective therapeutic knockdown of a gene responsible for oxidative stress in the central nervous system. This study is the first to conclusively demonstrate both siRNA-carrier delivery and therapeutic efficacy in any CNS disease model where the BBB remains intact and thus offers new avenues for potential treatments of oxidative stress underlying neuroinflammation in a variety of neuropathologies that are currently refractory to existing therapies.

## Introduction

A common denominator in neurodegenerative disorders such as stroke, traumatic brain injury, Parkinson’s disease, Alzheimer’s disease, Huntington’s disease, multiple sclerosis, and amyotrophic lateral sclerosis, is the induction of oxidative stress triggering neuroinflammation (Guo et al., 2013; Kowal et al., 2013; Murray et al., 2013; Bennett et al., 2014; Rubiano et al., 2015; Whiteford et al., 2015; Chiurchiù et al., 2016; Global Burden of Disease Cancer Collabration et al., 2017; Ma et al., 2017). Stroke is one of the leading causes of death in North America (Xu et al., 2018; Statistics Canada, 2018; Rios, 2019). It is caused by the impairment of cerebral blood flow (ischemic stroke) or the rupture of blood vessels in the brain (haemorrhagic stroke). The interruption of oxygenation and metabolites results in neuronal death. Ischemic stroke results in acute and chronic inflammation, as a result of activation and polarization towards pro-inflammatory (M1) microglia and subsequent activation of the NADPH oxidase (NOX) enzyme. The NOX enzymes are the primary source of reactive oxygen species (ROS) and are expressed by microglia after ischemic stroke (Li et al., 2009; Cooney et al., 2013). During ischemic stroke NOX2, NOX3, and NOX4 isotypes modulates their expression depending on cell type and time post-injury. NOX4 has been in neurons, astrocytes, and microglia, while NOX3 has been identified only in neurons, and NOX2 is expressed in microglia and neurons. As a result of these findings NOX4, is thought to be responsible for the majority of oxidative stress observed in acute traumatic brain injury (Cooney et al., 2013). Animals deficient in NOX4 are strongly protected from ischemic stroke (Kleinschnitz et al., 2010; Radermacher et al., 2013). A growing list of modalities are enabling pretreatment of acute ischemic stroke patients with a history of stroke or patients at risk for a stroke in attempt to reduce the severity of stroke when it occurs (Schmülling et al., 2003; Mackman, 2008; Fuentes et al., 2009; Milionis et al., 2009; Aoki and Uchino, 2011; Yoo, 2016; Qin et al., 2017; Tsivgoulis et al., 2018). Thus, approaches that limit stroke-related damage are emerging as important therapeutic tools.

The utility of short antisense/interfering RNA (siRNA) therapeutic approaches for central nervous system (CNS) diseases, such as stroke has been limited by their biodistribution *in vivo*. to the site of tissue damage alone. On their own, siRNA preferentially localizes to the kidney and liver, and their exclusion from the brain continues to hamper their potential and this represents a significant technical hurdle (Braasch et al., 2004). However, if brain delivery was possible, we hypothesized that reducing the expression of genes in the brain that potentiate the pathophysiology of stroke could be neuroprotective. NADPH oxidase type 4 (NOX4; Uniprot Q9JHI8) was chosen because this protein is known to be up-regulated during acute ischemic stroke, it is a major source of oxidative stress leading to neuronal apoptosis (Radermacher et al., 2013; Qin et al., 2017; Yao et al., 2017).

We have previously shown that melanotransferrin (MTf; Uniprot P08582), a mammalian iron-transport protein (Food et al., 1994; Rothenberger et al., 1996; Demeule et al., 2002; Moroo et al., 2003; Yang et al., 2004), can deliver anti-cancer drugs that reduce the *in situ* growth of brain tumours (Karkan et al., 2008). It has been shown that a 12 amino acid peptide derived from MTf (MTfp) can also act as a nanomule (i.e., a tiny conveyor or ferry for drugs) to deliver a protein-based analgesic to the brain (Thom et al., 2019). Here we report the creation of a fully functional peptide-oligonucleotide conjugate (POC) composed of MTfp and NOX4-targeted siRNA. Our data support the utility of MTfp-targeted delivery of siRNA to the brain as an efficacious pretreatment approach to reduce pathologies of the brain and the CNS.

## Methods and Materials

### Animal Experiments

All protocols and procedures involving the care and use of animals in these studies were reviewed and approved by the Animal Care Committees of Ottawa National Research Council of Canada or the University of British Columbia; both of which are governed by the Canadian Council of Animal Care. The mice were kept on a 12 h light-dark cycle and had food *ad libitum*.

For the delivery of siRNA to the brain experiments, 6–8 week-old female BALB/c mice (Jackson Laboratory; #000651) (16–20 g in weight) were used. For the induction of stroke experiments, 10 week-old male C57Bl/6 mice (Jackson Laboratory; #000664) were used.

### Synthesis of MTfp Conjugates

**FIGURE 1:**
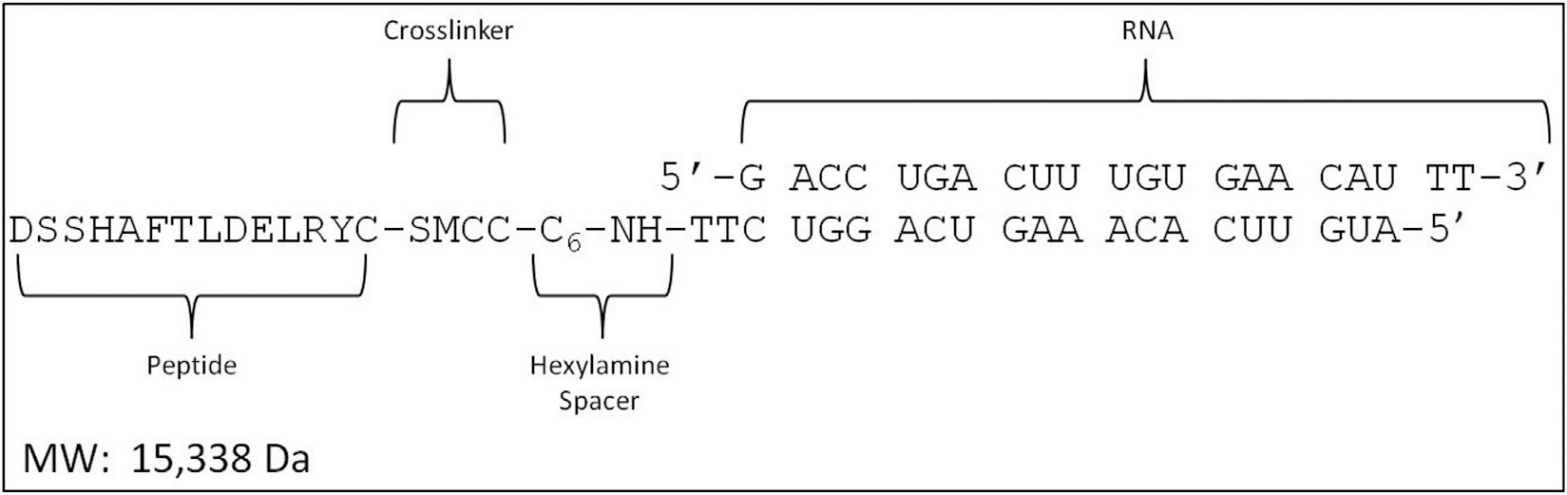
Design of the MTfp-RNA conjugates. SMCC, succinimidyl 4-(N- maleimidomethyl) cyclohexane-1-carboxylate.

MTfp, NOX4 siRNA (5ʹ-GAC​CUG​ACU​UUG​UGA​ACA​U-3ʹ), scrambled NOX4 siRNA (scramRNA, 5ʹ-GUA​AAU​UCU​CGC​GAC​UAG​U-3ʹ) and conjugates thereof were synthesized by Biosynthesis Inc. (Lewisville, United States). MTfp and RNA were produced independently and then chemically conjugated using the crosslinker succinimidyl-4-(N-maleimidomethyl) cyclohexane-1-carboxylate (Figure 1). High performance liquid chromatography and mass spectrometry indicated a purity of greater than 94%. The final products were lyophilized and stored at −80°C.

### 3D Fluorescence Microscopy Imaging

To determine the extent of delivery of RNA to the brain, 6–8 week-old female BALB/c mice (16–20 g in weight) received third IV injections at 1 h intervals with 0.1 ml/mouse PBS, siRNA_AF680_ (30 mg/kg) or MTfp-siRNA_AF680_ (30 mg/kg). Two hours post injection, mice received a second IV injection of tomato lectin conjugated to FITC (100 μg/mouse, 0.1 ml). The bloodstream was flushed by intracardiac perfusion for 10 min at a flow rate of 1 ml/min with heparinized saline (0.9% NaCl, 100 U/ml heparin), until the extremities appeared white, as we have done previously (Ujiie et al., 2003; Karkan et al., 2008).

The mice were then sacrificed and the brains were removed and fixed with 4% paraformaldehyde overnight and then transferred into PBS + 0.01% sodium azide and stored at 4°C. The brains were embedded in 4% agarose, fixed onto the microtome stage and sectioned (20 µm) at 4°C. Sections of the cerebral cortex were stained with DAPI and then mounted on microscopic slides. Glass coverslips were mounted on the sections using Prolong Gold antifade reagent.

The imaging experiments, which quantitatively assessed the ability of MTfp to direct the delivery of a therapeutic compound (NOX4 siRNA) to the brain as well as various localization issues, were performed using the Leica SP8 X system (Leica, Germany). 3D confocal images were acquired with a Leica AOBS SP8 laser scanning confocal microscope (Leica, Heidelberg, Germany) using a high-resolution Leica 63X/1.4 or 40X/1.3 Plan-Apochromat oil immersion objective lens. Excitations were performed using either diode or tunable white light laser sources. All images and spectral data (except DAPI) were generated using the highly sensitive HyD detectors (with gated option) in de-scanned mode. The backscattered emission signals from the sample were delivered through the tunable filter (AOBS), the detection pinhole, spectral dispersion prism, and finally to the PMT/HyD detectors. For 3D image data set acquisition, the excitation beam was first focused at the maximum signal intensity focal position within the brain tissue sample and the appropriate HyD gain levels were then selected to obtain the pixel intensities within range of 0–255 (8-bit images) using a color gradient function. The beginning and end of the 3D stack were set based on the signal level degradation. Series of 2D images for a selected 3D stack volume were then acquired with 1024 × 1024 pixels. The 3D stack images with optical section thickness (*z*-axis) of approximately 0.3 µm were captured from tissue volumes.

For each tissue volume reported here, z-section images were compiled and finally the 3D image restoration was performed using VOLOCITY software (Perkin Elmer, United Kingdom). The volume estimation was performed on the 3D image data sets recorded from four or more either cortical or hypothalamus areas of brain tissue samples. Algorithms were developed to automate the quantification of the test articles, blood capillaries and to accurately quantify test articles localized in blood capillaries vs. brain parenchyma. In these procedures, a noise removal filter (either Gaussian or kernel size of 3 × 3) was used to remove the noise associated with the images. To define the boundary between the objects (for instance, blood capillaries) and the background, the lower threshold level in the histogram was set to exclude all possible background voxel values. The sum of all the voxels above this threshold level is determined to be the volume. Fields from each experimental group were pooled. ANOVA was used to assess the volume fractions in the brain parenchyma (Prism 6, Graphpad, La Jolla, United States). The data were log transformed to meet the assumption of homogeneity of variance. Post hoc analysis used a Dunnett’s Test for multiple comparisons. The level of significance was *p* < 0.05 for the ANOVA and the *p*-values were corrected for multiple comparisons in the post hoc analysis.

### RNA Isolation and qPCR

Total RNA was isolated using TRIzol® reagent (Invitrogen, Burlington, Canada) from frozen brains of mice that had been treated with PBS, NOX4 siRNA, MTfp-NOX4 siRNA or MTfp-scramRNA. RNA was reverse transcribed to cDNA with a Script cDNA SuperMix agent (QuantaBio, Gaithersburg, United States) using random hexamers and oligo dT. SYBR® Green PCR Master Mix and RT-PCR Reagents kit (Applied Biosystems, Foster city, United States) were used to do quantitative real time PCR (qPCR) on an ABI 7500 FAST REAL TIME PCR System (Applied Biosystems, Foster city Unites States). Nox4 primers (5ʹ-CTT​GAC​TTG​ATG​GAG​GCA​GTA​G-3ʹ and 5ʹ-GCC​TTT​ATT​GTG​CGG​AGA​GA-3ʹ) and GAPDH primers (5ʹ-AAC​TTT​GGC​ATT​GTG​GAA​GG-3ʹ and 5ʹ-ACA​CAT​TGG​GGG​TAG​GAA​CA-3ʹ) were used. The master reaction mixture consisted of 1X SYBR®Green PRC buffer, 3 mM MgCl_2_, 1 mM dNTP, 0.625 U Taq polymerase, 0.25 U amperase UNG, 10 ng cDNA, and 300 nM primers (sense and antisense). The qPCR was carried out at 50°C for 2 min, 95°C for 10 min, followed by 40 cycles at 95°C for 15 s, and 60°C for 1 min. The qPCR efficiency was determined for each gene with the slope of a linear regression model, and all samples displayed efficiencies between 84 and 96%. Negative controls containing no target RNA were included with each of the qPCR runs. The qPCR values were compared using the method described by Huggett et al. (Huggett et al., 2005). Data are expressed as mean ± standard error of mean. In multiple group comparisons, data were analyzed by one-way ANOVA with Tukey post-hoc test. *p-*values less than 0.05 were considered significant.

### Stroke Induction by Transient Middle Cerebral Artery Occlusion

Work with the mouse stroke model was performed at the National Research Council (Ottawa, Canada) and at the University of British Columbia (Vancouver, Canada), by different operators. Male C57Bl/6 mice (10 weeks old) were injected by IV three times, at 1 h intervals, with PBS, NOX4 siRNA, MTfp-siRNA or MTfp-scram RNA (30 mg/kg). One hour after the last injection, an ischemic stroke was induced on the left hemisphere by MCAO. The external and internal carotid arteries (ECA and ICA, respectively), were dissected and isolated and the common carotid artery (CCA) ligated. A nylon thread coated with dental silicon (Cat. # 60231012, Doccol Corp) was inserted into a small hole in the ECA towards the bifurcation of the CCA. The thread was gently introduced into the ICA, passed into the Circle of Willis thus occluding the opening of the middle cerebral artery. Body temperature was not allowed to drop below 36°C and anesthesia was maintained throughout the surgery occlusion. The filament was removed after 1 h, and the ligature on the CCA was removed allowing reperfusion of the affected brain area. Mice were sacrificed at 24 h post reperfusion. Sham surgeries (arteries isolated but not occluded) on animals that received all three injections of PBS were also performed as controls and animals were under anesthesia for as long as the stroke-induced animals.

### Stroke Analysis

An individual, who was blinded to the treatment each mouse had received, assessed post stroke behaviour. Mice were assessed at 0.5 and 24 h after recovery from anesthesia using a method described by Jiang *et al* (Jiang et al., 2005). A score of zero is normal; one is mild turning behaviour with or without inconsistent curling when picked up by tail and <50% attempts to curl to the contralateral side; two is mild consistent curling with >50% attempts to curl to contralateral side; three is strong and immediate consistent curling, mouse holds curled position for more than 1–2 s, the nose of the mouse almost reaches tail; four is severe curling progressing into barrelling, loss of walking or righting reflex; five is comatose or moribund. According to this method, the neuroscore at 0.5 h is a measure of surgical success and identifies animals that had received abnormally severe or mild damage during the procedure. Mice were excluded from the final calculation for having a neurological scores below 1 (no effective stroke) or above 2.5 (surgical trauma).

To determine infarct size, mice were sacrificed after the second behavioural assessment (24 h post-op). Mice were terminally sedated with 0.2 ml of ketamine (200 mg/kg) and Rhompun (20 mg/kg) and perfused transcardially at 1 ml/min for 10 min with heparinized saline (0.9% NaCl, 100 U/ml heparin). Brains were removed, sectioned and stained with 2% TTC in PBS to visualize the infarcts. Images of the brain slides were obtained after 15–20 min staining at room temperature. The brain slices were then cut in half to separate the ischemic hemisphere and the contralateral hemisphere. Tissue was rinsed with physiological saline and then exposed to a mixture of ethanol/dimethyl sulfoxide (1:1) for 24 h in the dark to solubilise the coloured formazan product. The absorbance at 485 nm was measured. Percentage loss in brain TTC staining in the ischemic side of the brain was compared with the contralateral side of the brain of the same animal using the following equation.

$$\mathrm{\% loss = 100 x}\;\left(1-\frac{\mathrm{absorbance: ischemic hemisphere}}{\mathrm{absorbance: contralateral hemisphere}}\right)$$

Data are expressed as mean ± standard error of mean. In multiple group comparisons, data were analyzed by one-way ANOVA with Tukey post-hoc test. *p-*values less than 0.05 were considered significant.

## Results

### MTfp-NOX4 siRNA Reduces Stroke Damage

**FIGURE 2:**
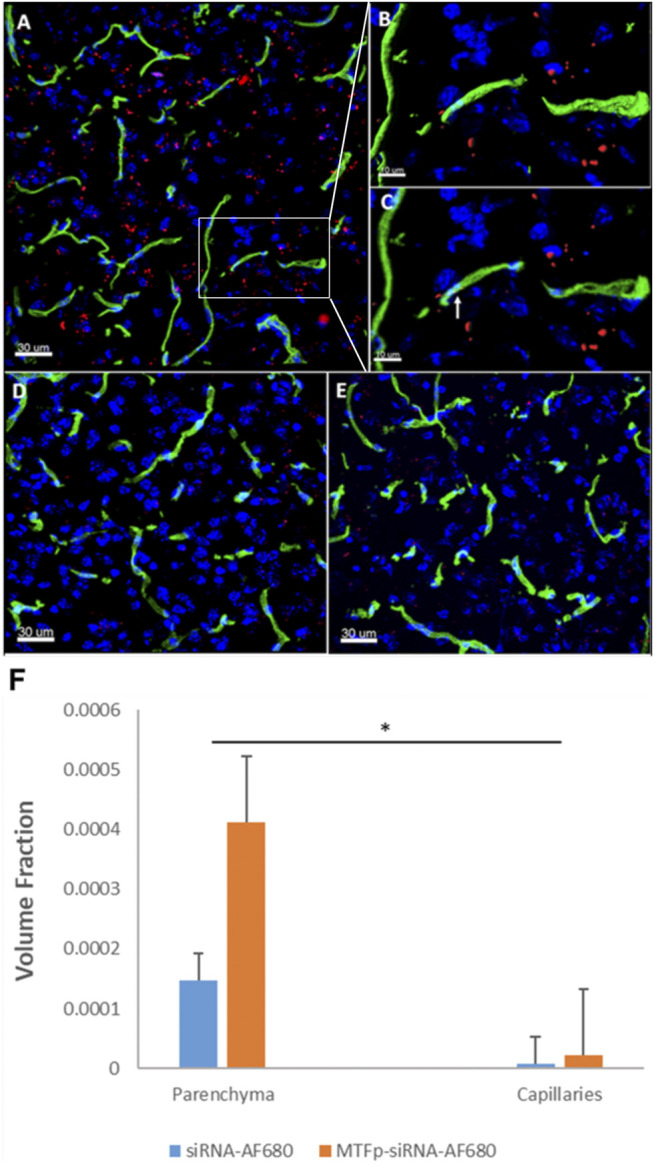
Conjugation to MTfp enables siRNA to cross an intact BBB and into the brain parenchyma. Representative 3D confocal images showing localization of MTfp-siRNA in the brain of mice with intact BBB. Cell nuclei are blue (DAPI) and capillaries are green (Tomato lectin-FITC). **(A)** AF680 fluorescence (red) in the cerebral cortex sections from a mouse treated with MTfp-siRNA_AF680_; **(B)** Shows the enlarged area that has been surface-rendered to indicate the surface of the capillaries where FITC labelled capillaries (green) and MTfp-siRNA_AF680_ (red); **(C)** Shows the enlarged area where MTfp-siRNA_AF680_ is localized within the blood capillaries (yellow dots, see arrow); (**D)** AF680 fluorescence (red) in the mouse cerebral cortex treated with PBS (i.e., background fluorescence); **(E)** Control AF680 fluorescence (red) in the mouse cortex treated with siRNA_AF680_. **(F)** Distribution of MTfp-siRNA in the cortex of wild type mice with intact BBB. Values indicate total AF680 fluorescence normalized to total tissue volume (VTA in Supplementary Table S1) and then normalized to the total AF680 fluorescence seen in PBS (background). Data are represented as means ± SD (*n* = 3, eight fields of view per animal). *p-value < 0.05.

We have described the identification and transport characteristics of the 12 amino acid MTfp elsewhere (Thom et al., 2019). To assess whether MTfp could deliver RNA cargo to the CNS, MTfp was conjugated to AF680-conjugated NOX4-specific siRNA (Figure 1) and injected IV into mice. The brains were then examined by 3D confocal fluorescence microscopy (Figure 2) to determine whether MTfp could enable increased delivery of the siRNA across the BBB (i.e., the vasculature) to the brain parenchymal tissues (Figures 2A–C). We found the conjugation of MTfp to siRNA allowed increased localization of siRNA into the brain parenchyma compared to siRNA alone (Figure 2A vs. 2D and Supplementary Table S1). Interestingly, a small amount of siRNA was able to cross the BBB (Figure 2F).

**FIGURE 3:**
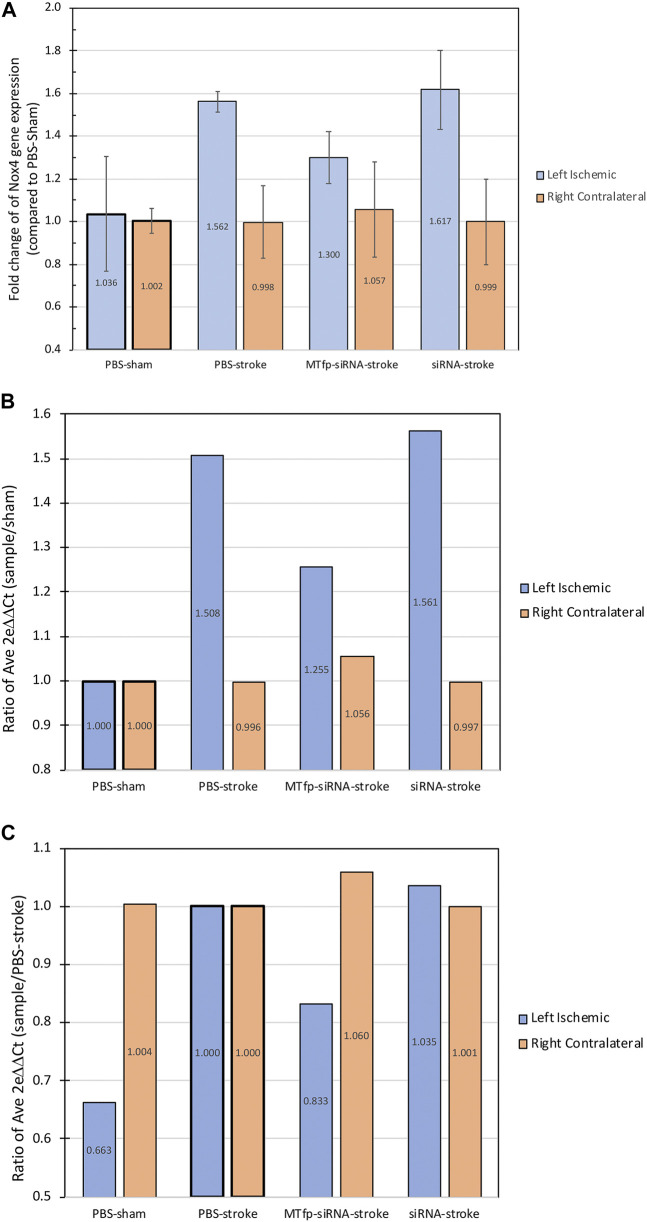
NOX4 mRNA expression in the brain after siRNA treatment. The graphs show the fold change of Nox4 gene expression in the mouse brain hemispheres measured by qPCR following treatment and 24 h post-stroke, showing both the expression in left ischemic side and the right contralateral side. **(A)** Values are normalized to *β*-actin gene expression and individual samples are compared to the corresponding average PBS-sham condition (ischemic or contralateral). Data are shown as mean ± SEM. (*n* = 2–4 mice per group). **(B)** Ratio of average fold change of each group compared to PBS-sham. **(C)** Ratio of average fold change of each group compared to PBS-stroke. “Left ischemic” in blue, refers to the measurments in the left stroke-induced hemisphere and “Right contralateral” in orange, refers to the measurement in the right non-stroke hemisphere. Raw data and calculations are shown in Supplementary Table S3.

To demonstrate the therapeutic efficacy of cargo delivery to the CNS by MTfp, MTfp conjugated to NOX4-specific siRNA was examined in mouse model of ischemic stroke. Mice were dosed three times, at 1 h intervals, and then subjected to ischemic stroke in the left hemisphere for a duration of 1 h by middle cerebral artery occlusion (MCAO). In this proof-of-concept study, we chose to administer treatment (MTfp-siRNA, MTfp-scramRNA, NOX4 siRNA alone, and PBS controls) prior to stroke induction to demonstrate delivery across the intact BBB since the integrity will certainly be compromised by the invasive nature of stroke induction. Furthermore, this therapy is intended to reduce NOX4/hypoxia associated pathology and will have no effect if blood flow to the site of injury is impaired (i.e., blood clots or filament occlusion). Animals were sacrificed at 24 h post reperfusion. It is known that induction of ischemic stroke leads to elevated CNS expression of NOX4 (Kleinschnitz et al., 2010). This trend is seen in experiments, conducted by a different operators, in animals induced with stroke after being treated with PBS, NOX4 siRNA alone (Figure 3 and Supplementary Table S3) or MTfp-scramRNA control (Supplementary Tables S1, S2, S4). However, when treated with MTfp-siRNA, NOX4 up-regulation was reduced after stroke, where NOX4 mRNA was only 80% of the levels observed with the PBS stroke-induced control (Figure 3).

**FIGURE 4:**
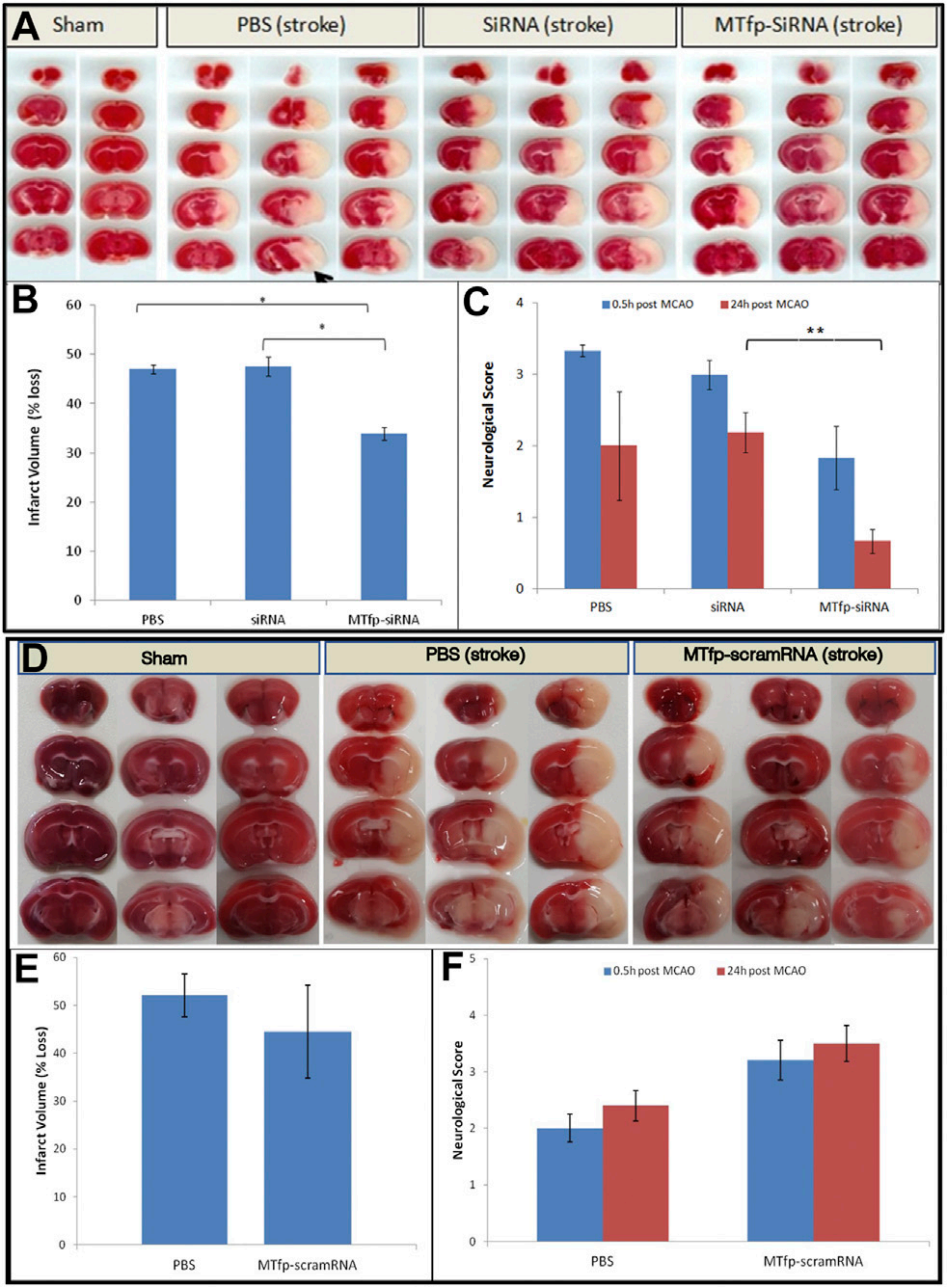
Top panel. **(A** *–* **C)**: Treatment with MTfp-siRNA confers neuroprotection and reduces damage after ischemic stroke. **(A)** TTC-stained brain sections of mice receiving various IV injections, followed by ischemic stroke. Tissue was collected 24 h after surgery **(B)** Infarct volume was quantitated by measuring absorbance of solvent extracted dye (* *p* < 0.05, one-way ANOVA). **(C)** Neuroscore at 0.5 and 24 h after stroke induction in mice pretreated with siRNA, MTfp-siRNA or PBS control. *p < 0.05, **p < 0.001. Bottom Panel **(D–F)**: Treatment with MTfp-scrambled RNA does not offer neuroprotection after ischemic stroke. **(D)** TTC-stained brain sections of mice receiving various IV injections, followed by ischemic stroke. **(E)** Infarct volume was quantitated by measuring absorbance (485 nm) of solvent extracted dye. Data are shown as mean ± SEM (no significant difference). **(F)** Neuroscore at 0.5 and 24 h after stroke induction. Data are shown as mean ± SEM (There is no significant difference between the treatments at either time point) (*n* = 3 mice per group).

Finally, we assessed whether MTfp-siRNA mediated NOX4 knockdown resulted in protection against neurological deterioration. Mice were treated and subjected to ischemic stroke as described above. Neuroscore (Jiang et al., 2005) was measured 0.5 h after removal of anesthesia (to determine surgical efficiency) and again at 24 h post stroke (to measure recovery and outcome). After the second behavioural assessment, animals were sacrificed and infarct volume was quantitated by 2,3,5-triphenyltetrazolium chloride (TTC) staining. Total infarct volume as well as motor deficits were measured (Figure 4; Supplementary Table S2). Compared to PBS controls, sham surgery, siRNA alone and MTfp-scramRNA controls, the MTfp-siRNA resulted in significantly smaller infarcts (Figure 4) and corresponded to less severe paralysis immediately after cessation of MCAO (Figure 4). Twenty-four hours after surgery, mice that had been treated with MTfp-siRNA achieved neurological scores of <1 which is functionally near normal, while the control animals remained mentally and physically impaired.

## Discussion

We chose to focus on stroke because it is a leading cause of death worldwide, and is a difficult disease to treat. The gene, NOX4, has been shown to be upregulated during stroke and thus NOX4 offers a convenient target to test the therapeutic effects of a MTfp-delivered siRNA. Currently, recombinant tissue plasminogen activator is the only approved drug for treatment of stroke but due to contraindications, it can only be used in about 10% of patients. Furthermore, mainstream approaches are turning to pretreament approaches that stabilize and reduce the brain damage in patients with recurrent stroke (Schmülling et al., 2003; Mackman, 2008; Fuentes et al., 2009; Milionis et al., 2009; Yoo, 2016; Tsivgoulis et al., 2018) or patients at a risk for stroke (Aoki and Uchino, 2011). Thus, approaches that limit stroke-related damage are emerging as important therapeutic tools.

New and effective treatments are needed for reducing the frequency and severity of recurrent stroke. Oxidative stress, which is the damage caused by reactive oxygen species, appears to play a central role in neuronal death in stroke. NOX4 is one of the major sources of oxidative stress and therefore an excellent therapeutic target in acute stroke. Upon ischemia, NOX4 is highly induced in both human and mouse brains. In addition, following cerebral ischemia, mice genetically deficient in NOX4 (Nox4^−/−^) are largely protected from oxidative stress, neuronal death and BBB leakage (Kleinschnitz et al., 2010). Furthermore, NOX4 is a culprit in CNS disease besides stroke and therefore this POC may have utility in other brain pathologies (Kim et al., 2016). However, targeting NOX4 in the brain is challenging due to the difficulty in getting a therapeutic across the BBB. Therefore, we used the small peptide, MTfp, as a brain-targeting carrier for NOX4 siRNA.

Our data, obtained using a murine stroke model with the POC targeted against NOX4 for the amelioration of stroke is an example of efficacious gene knockdown in a model of a disease resident in the brain in which the BBB is intact. In this proof-of-concept study we opted to deliver the treatment before stroke induction to assess this approach in reducing the severity of stroke. This was also done to show that MTfp can cross an intact BBB, since stroke, by definition, results in a disruption of the BBB. Using a novel stable isotopic liquid chromatography mass spectrometry methodology to measure the transport of MTfp into the brain, we have reported elsewhere the initial rate of entry as 2.1 nM/min (Eyford et al., 2019). Additionally, stroke impairs blood flow and it is possible that if therapies were to be administered IV during or after a stroke, they would be less likely to gain access to the affected tissues. We suspect that, to achieve best outcomes, the POC conjugate described here will have to be used in combination with traditional fibrolytic therapies. Despite the clinical limitations, the POC used in this study demonstrates that MTfp has the capability of granting access to the CNS, to a molecule (RNA) that would normally be excluded.

Previous attempts to deliver siRNA to the CNS have relied on antibody-targeted liposomes (Zhang et al., 2004) or non-covalent, electrostatic interactions with a peptide derived from rabies virus glycoprotein (Kumar et al., 2007; Kim et al., 2010; Alvarez-Erviti et al., 2011) or by intracerebroventricular injection into the brain (Helmschrodt et al., 2017). Free siRNA, and even many lipid-encapsulated formulations, are quickly eliminated from the circulatory system to accumulate within organs, such as kidney and liver, and degraded by the mononuclear phagocyte system (Park et al., 2016). Furthermore, nanotechnology-based siRNA carriers such as protease-responsive, brain targeting nanoparticles (Guo et al., 2018) and others approaches (Mann et al., 2016; Tian et al., 2018), simply localise to the area of tissue damage and do not transcytosis across the BBB and thus have limited biodistribution. Thus they never reach the CNS in significant amounts to have a biological outcome, and this may lead to poor efficacy in a clinical setting. Some examples exist for the entry of free siRNA (Xie et al., 2020) or siRNA-encapsulated nanoparticles (Li et al., 2021) to enter the CNS, however, to date these intriguing approaches are only described to exert their therapeutic effect after a prior breach of the BBB and do not directly show transcytosis of the intact BBB. For example, some studies on traumatic brain injury indicate that BBB may be breached for several days after induction of trauma (Li et al., 2021), others indicate the BBB is permeable for weeks after initial injury (e.g., ischemia, blunt force trauma) (Strbian et al., 2008; Price et al., 2016), and some BBB tight junction components can be downregulated for up to 8 weeks (Nag et al., 2007). In addition, observation of brain penetration in Alzheimer’s disease and Multiple Sclerosis models have shown a prior breach in the BBB as part of the pathogenesis of the disease (Ujiie et al., 2003; Bennett et al., 2010; Biron et al., 2011; Biron et al., 2013), and siRNA studies in these models similarly cannot be used to conclude transcytosis of siRNA across an intact BBB (Zhou et al., 2020). This is the scientific background that provided the premise for the current study. In this study the BBB is intact at the time of injection of the “nanomule” siRNA conjugate into the peripheral circulatory system and accumulates in significant enough quantities to ameliorate the pathogenic features of ischemic stroke after it is subsequently induced. In contrast, the POC we describe, MTFp, is connected covalently and can be produced using standard peptide/RNA synthesis and conjugation technologies. We have shown in another study that MTfp accumulates within the brain paranchyma at a rate of 2.1 nM/min after delivery, and that after only 30 min, the accumulation of MTfp within the brain was at a ratio of ¼ of that found in the kidneys, which is extremely efficient for drug delivery across the BBB (Eyford et al., 2019). The MTfp is derived from a protein of human origin, further reducing the likelihood of immunogenicity compared to a viral carrier. It traverses the intact BBB, transports without degradation, to be widely distributed in microglia, neurons and astroglia in the CNS, and is delivered in sufficient quantities to knockdown NOX4 and alleviate ischemic stroke. This approach represents a less invasive method for delivery of siRNA and other drug conjugates to the CNS and could conceivably be used as a platform to modify a number of diseases in the brain.

In conclusion, we describe the modulation of neuroinflammation in the central nervous system using an siRNA directed at NOX4 expressed in the resident brain cells. This discovery will be useful for understanding the mechanism leading to neuroinflammatory diseases such as stroke, and perhaps lead to pretreatment and post-stroke regimens. In addition, this work identifies a novel, non-invasive general method of ferrying therapeutic cargo across the BBB to dampen the oxidative stress triggering neuroinflammation that leads to many neuropathologies. Future focus will be on expanding the utility of this new carrier to traverse therapeutics across the battlement of the BBB for the treatment of other pathologies within the physiological fortress of the CNS.

## Data Availability

The original contributions presented in the study are included in the article/Supplemental Material, further inquiries can be directed to the corresponding authors.
