# Corrigendum: A Nanomule Peptide Carrier Delivers siRNA Across the Intact Blood-Brain Barrier to Attenuate Ischemic Stroke

**DOI:** 10.3389/fmolb.2021.687587

**Published:** 2021-05-07

**Authors:** Brett A. Eyford, Chaahat S. B. Singh, Thomas Abraham, Lonna Munro, Kyung Bok Choi, Tracy Hill, Rhonda Hildebrandt, Ian Welch, Timothy Z. Vitalis, Reinhard Gabathuler, Jacob A. Gordon, Hans Adomat, Emma S.T. Guns, Chieh-Ju Lu, Cheryl G. Pfeifer, Mei Mei Tian, Wilfred A. Jefferies

**Affiliations:** ^1^Michael Smith Laboratories, University of British Columbia, Vancouver, BC, Canada; ^2^The Vancouver Prostate Centre, Vancouver General Hospital, Vancouver, BC, Canada; ^3^Centre for Blood Research, University of British Columbia, Vancouver, BC, Canada; ^4^The Djavad Mowafaghian Centre for Brain Health, University of British Columbia, Vancouver, BC, Canada; ^5^Department of Medical Genetics, University of British Columbia, Vancouver, BC, Canada; ^6^Department of Neural and Behavioral Sciences and Microscopy Imaging Core Lab, Pennsylvania State College of Medicine, Hershey, PA, United States; ^7^Centre for Comparative Medicine, University of British Columbia, Vancouver, BC, Canada; ^8^Department of Chemistry, University of British Columbia, Vancouver, BC, Canada; ^9^Bioasis Technologies Inc., Guilford, CT, United States; ^10^King's College London, London, United Kingdom; ^11^Department of Urologic Sciences, University of British Columbia, Vancouver, BC, Canada; ^12^Department of Microbiology and Immunology, University of British Columbia, Vancouver, BC, Canada; ^13^Department of Zoology, University of British Columbia, Vancouver, BC, Canada

**Keywords:** stroke, peptide-oligonucleotide conjugate, MTfp, blood-brain barrier, NOX4, siRNA

In the original article, there was an error in the Funding statement. “MT” should be removed from “BTI MT.” The corrected Funding statement should read:

“Funding for this work was provided to WJ in the Michael Smith Laboratories, at the University of British Columbia and the Vancouver Prostate Centre, at Vancouver General Hospital, by a grant from the Canadian Institutes for Health Research (MOP-133635), a grant from the Natural Sciences and Engineering Research Council of Canada (CRDPJ 452456–13) in collaboration with Bioasis Technologies Inc. (BTI, meimei@bioasis.us), a grant from the W. Garfield Weston Foundation (RR161038), and donations from the Sullivan Urology Foundation at Vancouver General Hospital. TA was supported by NIH grants 1S10OD010756–01A1 and 1S10OD018124–01A1. BE was supported by Postdoctoral Fellowships from the Michael Smith Foundation for Health Research, the Centre for Blood Research at the University of British Columbia and the Pacific Alzheimer's Foundation. CS was supported by an Alzheimer's Drug Discovery Foundation Outstanding Young Investigator Scholarship, and by a William and Dorothy Gilbert Graduate Scholarship in Biomedical Sciences at the University of British Columbia and a scholarship from the Centre for Blood Research Graduate Student Award at the University of British Columbia.”

Additionally, there was a mistake in the COI section, which should now read:

“The authors declare that this study received funding from Bioasis Technologies Inc. The funder did not influence the study design, collection, analysis, interpretation of data, the writing of this article or the decision to submit it for publication.”

The authors apologize for these errors and state that this does not change the scientific conclusions of the article in any way. The original article has been updated.

