# Supplementary material for: A Nanomule Peptide Carrier Delivers siRNA Across the Intact Blood-Brain Barrier to Attenuate Ischemic Stroke
Source: Front Mol Biosci. 2021 Mar 26;8:611367. doi: 10.3389/fmolb.2021.611367 (PMC8044710; doi:10.3389/fmolb.2021.611367)
Supplement: Supplementary file 1 [file datasheet1.pdf]

Supplemental Information for: "A Nanomule Peptide Carrier Delivers siRNA Across the Blood-Brain Barrier to Attenuate Neuroinflammation in Ischemic Stroke"

Authors:

Brett A. Eyford[1,2,3,4+], Chaahat S.B. Singh[1,2,3,4,5+], Thomas Abraham[6+], Lonna Munro[1,3], Kyung Bok Choi[1,3,] Tracy Hill[7], Rhonda Hildebrandt[7], Ian Welch[7], Timothy Z. Vitalis[9], Reinhard Gabathuler[9,10], Jacob A. Gordon[2,11], Hans Adomat[2,11], Emma S.T. Guns[2,11], Chieh-Ju Lu[1,2,3,4], Cheryl G. Pfeifer[1,2,3,4], Mei Mei Tian[9], Wilfred A. Jefferies[1,2,3,4,5,11,12,13*]

Affiliations:

[1]Michael Smith Laboratories, University of British Columbia, 2185 East Mall, Vancouver, BC, Canada, V6T 1Z4.

[2]The Vancouver Prostate Centre, Vancouver General Hospital, 2660 Oak Street, Vancouver, BC, Canada, V6H 3Z6.

[3]Centre for Blood Research, University of British Columbia, 2350 Health Sciences Mall, Vancouver, BC, Canada, V6T 1Z4.

[4]The Djavad Mowafaghian Centre for Brain Health, University of British Columbia, 2215 Wesbrook Mall, Vancouver, BC, Canada, V6T 1Z4.

[5]Department of Medical Genetics, University of British Columbia, 2350 Health Sciences Mall, Vancouver, BC, Canada, V6T 1Z4.

[6]Department of Neural and Behavioral Sciences and Microscopy Imaging Core Lab, Pennsylvania State College of Medicine, 500 University Drive, Hershey, PA, USA, 17033-2390.

[7]Centre for Comparative Medicine, University of British Columbia, 4145 Wesbrook Mall, Vancouver, BC, Canada, V6T 1W5

[8]Department of Chemistry, University of British Columbia, 2350 Health Sciences Mall, Vancouver, BC, Canada, V6T 1Z4.

[9]Bioasis Technologies Inc., 14 Water Street, Guilford, CT 06437, USA.

[10]King's College London, London, UK

[11]Department of Urologic Sciences, University of British Columbia, Gordon & Leslie Diamond Health Care Centre, Level 6, 2775 Laurel Street, Vancouver, BC CanadaV5Z 1M9

[12]Department of Microbiology and Immunology, University of British Columbia, 2350 Health Sciences Mall, Vancouver, BC, Canada, V6T 1Z4.

[13]Department of Zoology, University of British Columbia, 6270 University Blvd., Vancouver, BC,

Supplemental Table 1. Fluorescence associated with the localization of siRNA and MTfp-siRNA to brain tissues.

Legend:

TTV - Total Tissue Volume

- Sum of voxels in entire reconstructed 3D field

BCV - Brain Capillary Volume

- Total FITC voxels

BPV - Brain Parenchyma Volume

- TTV minus BCV

TTAV - Total Test Article Volume

- Total voxels of AF680 fluorescence

TAV_{BC} – Volume of fluorescence in Brain Capillaries

- Voxels of AF680 Co-localized with FITC voxels

TAV_{BP} - Volume of fluorescence in Parenchyma

- TTAV minus TAV_{BC}

VTA_{BC} - Volume fraction of Test Article in Brain Capillaries

- TAV_{BC} / TTV

VTA_{BP} - Volume fraction of Test Article in Brain Parenchyma

- TAV_{BP} / TTV

VTA_{TOT} – Volume fraction of Test Article in the Brain Capillaries and Parenchyma

- TTAV / TTV 66

Supplemental Table 1. Volume fluorescence associated with the localization of siRNA and MTfp-siRNA to brain tissues

Animal ID (Treatment Group)	Brain Region	Field	TTV (µm³)	BCV (µm³)	BPV (µm³)	TTAV (µm³)	TAV$_{BC}$ (µm³)	TAV$_{BP}$ (µm³)	VTA$_{BC}$	VTA$_{BP}$	VTA$_{TOT}$
M1 (PBS)	Cerebral Cortex	1	1.83E+06	1.86E+04	1.81E+06	695	32	663	1.75E-05	3.63E-04	3.80E-04
	Cerebral Cortex	2	1.83E+06	2.01E+04	1.81E+06	443	7	436	3.67E-06	2.39E-04	2.43E-04
	Cerebral Cortex	3	1.83E+06	2.29E+04	1.80E+06	691	8	683	4.32E-06	3.74E-04	3.79E-04
	Cerebral Cortex	4	1.83E+06	2.26E+04	1.80E+06	257	9	248	5.01E-06	1.36E-04	1.41E-04
**Mean (PBS)**									7.62E-06	2.78E-04	2.86E-04
**SEM (PBS)**									3.30E-06	5.65E-05	5.81E-05
M2 (siRNA-AF680)	Cerebral Cortex	1	1.83E+06	2.44E+04	1.80E+06	333	29	304	1.59E-05	1.66E-04	1.82E-04
	Cerebral Cortex	2	1.83E+06	3.21E+04	1.79E+06	241	9	232	5.03E-06	1.27E-04	1.32E-04
	Cerebral Cortex	3	1.83E+06	2.00E+04	1.81E+06	734	35	699	1.92E-05	3.83E-04	4.02E-04
	Cerebral Cortex	4	1.83E+06	2.42E+04	1.80E+06	838	15	824	8.08E-06	4.51E-04	4.59E-04
	Thalamus	1	1.72E+06	1.67E+04	1.71E+06	1651	21	1630	1.23E-05	9.46E-04	9.59E-04
	Thalamus	2	1.72E+06	2.99E+04	1.69E+06	1158	17	1140	1.00E-05	6.62E-04	6.72E-04
	Thalamus	3	1.72E+06	2.24E+04	1.70E+06	1145	4	1142	2.05E-06	6.63E-04	6.65E-04
	Thalamus	4	1.72E+06	2.79E+04	1.69E+06	919	19	901	1.10E-05	5.23E-04	5.34E-04
M3 (siRNA-AF680)	Cerebral Cortex	1	1.68E+06	2.00E+04	1.66E+06	870	30	841	1.77E-05	5.01E-04	5.19E-04
	Cerebral Cortex	2	1.68E+06	3.05E+04	1.65E+06	1197	146	1051	8.69E-05	6.26E-04	7.13E-04
	Cerebral Cortex	3	1.68E+06	2.31E+04	1.65E+06	775	34	741	2.04E-05	4.41E-04	4.62E-04
	Cerebral Cortex	4	1.68E+06	2.71E+04	1.65E+06	1343	125	1218	7.47E-05	7.26E-04	8.01E-04
	Thalamus	1	1.72E+06	2.52E+04	1.70E+06	777	11	766	6.47E-06	4.45E-04	4.51E-04
	Thalamus	2	1.72E+06	3.31E+04	1.69E+06	616	12	604	6.81E-06	3.51E-04	3.58E-04
	Thalamus	3	1.72E+06	3.40E+04	1.69E+06	746	34	711	2.00E-05	4.13E-04	4.33E-04
	Thalamus	4	1.72E+06	2.30E+04	1.70E+06	346	11	336	6.10E-06	1.95E-04	2.01E-04
M4 (siRNA-AF680)	Cerebral Cortex	1	1.71E+06	2.49E+04	1.69E+06	849	16	834	9.10E-06	4.87E-04	4.96E-04
	Cerebral Cortex	2	1.71E+06	2.87E+04	1.68E+06	905	16	888	9.54E-06	5.19E-04	5.28E-04
	Cerebral Cortex	3	1.71E+06	2.68E+04	1.69E+06	827	14	813	8.24E-06	4.75E-04	4.83E-04
	Cerebral Cortex	4	1.71E+06	2.79E+04	1.68E+06	916	28	888	1.62E-05	5.19E-04	5.35E-04
	Thalamus	1	1.76E+06	2.90E+04	1.73E+06	467	12	455	7.00E-06	2.59E-04	2.66E-04
	Thalamus	2	1.76E+06	2.44E+04	1.73E+06	220	2	218	1.10E-06	1.24E-04	1.25E-04
	Thalamus	3	1.76E+06	3.14E+04	1.73E+06	191	17	175	9.57E-06	9.94E-05	1.09E-04
	Thalamus	4	1.76E+06	3.17E+04	1.73E+06	167	0	167	0.00E+00	9.53E-05	9.53E-05
**Mean (siRNA-AF680)**	Cerebral Cortex								2.43E-05	4.52E-04	4.76E-04
**SEM (siRNA-AF680)**	Cerebral Cortex								7.80E-06	4.85E-05	5.37E-05
**Mean (siRNA-AF680)**	Thalamus								7.70E-06	3.98E-04	4.06E-04
**SEM (siRNA-AF680)**	Thalamus								1.59E-06	7.67E-05	7.74E-05
M5 (MTFp-siRNA-AF680)	Cerebral Cortex	1	1.83E+06	2.56E+04	1.80E+06	1603	24	1578	1.33E-05	8.65E-04	8.78E-04
	Cerebral Cortex	2	1.83E+06	2.11E+04	1.80E+06	1698	47	1651	2.58E-05	9.04E-04	9.30E-04
	Cerebral Cortex	3	1.83E+06	1.56E+04	1.81E+06	1566	52	1513	2.87E-05	8.29E-04	8.58E-04
	Cerebral Cortex	4	1.83E+06	1.56E+04	1.81E+06	2580	718	1862	3.93E-04	1.02E-03	1.41E-03
	Thalamus	1	1.72E+06	1.61E+04	1.71E+06	1046	17	1028	1.01E-05	5.97E-04	6.07E-04
	Thalamus	2	1.72E+06	1.93E+04	1.70E+06	378	21	357	1.24E-05	2.07E-04	2.19E-04
	Thalamus	3	1.72E+06	1.06E+04	1.71E+06	1883	62	1821	3.59E-05	1.06E-03	1.09E-03
	Thalamus	4	1.72E+06	1.42E+04	1.71E+06	713	14	699	8.03E-06	4.06E-04	4.14E-04
M6 (MTfp-siRNA-AF680)	Cerebral Cortex	1	1.68E+06	1.89E+04	1.66E+06	691	7	684	4.31E-06	4.08E-04	4.12E-04
	Cerebral Cortex	2	1.68E+06	2.39E+04	1.65E+06	409	16	393	9.30E-06	2.34E-04	2.44E-04
	Cerebral Cortex	3	1.68E+06	1.62E+04	1.66E+06	1040	76	964	4.55E-05	5.75E-04	6.20E-04
	Cerebral Cortex	4	1.68E+06	2.14E+04	1.66E+06	564	106	457	6.35E-05	2.73E-04	3.36E-04
	Thalamus	1	1.77E+06	9.93E+03	1.76E+06	137	3	134	1.51E-06	7.58E-05	7.73E-05
	Thalamus	2	1.77E+06	1.04E+04	1.76E+06	261	8	254	4.34E-06	1.43E-04	1.48E-04
	Thalamus	3	1.77E+06	5.91E+03	1.76E+06	56	0	56	0.00E+00	3.17E-05	3.17E-05
	Thalamus	4	1.77E+06	1.35E+04	1.76E+06	217	1	217	5.07E-07	1.22E-04	1.23E-04
M7 (MTfp-siRNA-AF680)	Cerebral Cortex	1	1.70E+06	1.59E+04	1.68E+06	1028	6	1022	3.34E-06	6.02E-04	6.05E-04
	Cerebral Cortex	2	1.70E+06	1.53E+04	1.68E+06	479	3	476	1.70E-06	2.80E-04	2.82E-04
	Cerebral Cortex	3	1.70E+06	9.78E+03	1.69E+06	3220	1	3219	5.54E-07	1.89E-03	1.89E-03
	Cerebral Cortex	4	1.70E+06	1.29E+04	1.69E+06	677	20	657	1.18E-05	3.86E-04	3.98E-04
	Thalamus	1	1.72E+06	1.54E+04	1.71E+06	2054	13	2042	7.49E-06	1.19E-03	1.19E-03
	Thalamus	2	1.72E+06	2.22E+04	1.70E+06	2151	25	2126	1.43E-05	1.23E-03	1.25E-03
	Thalamus	3	1.72E+06	2.57E+04	1.70E+06	2197	11	2186	6.49E-06	1.27E-03	1.28E-03
	Thalamus	4	1.72E+06	2.74E+04	1.69E+06	3374	15	3358	8.84E-06	1.95E-03	1.96E-03
**Mean (MTfp-siRNA-AF680)**	Cerebral Cortex								5.01E-05	6.89E-04	7.39E-04
**SEM (MTfp-siRNA-AF680)**	Cerebral Cortex								3.17E-05	1.35E-04	1.44E-04
**Mean (MTfp-siRNA-AF680)**	Thalamus								9.15E-06	6.90E-04	6.99E-04
**SEM (MTfp-siRNA-AF680)**	Thalamus								2.76E-06	1.82E-04	1.83E-04

TTV - Total Tissue Volume (Sum of voxels in entire reconstructed 3D field)

BCV - Brain Capillary Volume (Total FITC voxels)

BPV - Brain Parenchyma Volume (TTV minus BCV)

TTAV - Total Test Article Volume (Total voxels of AF680 fluorescence)

TAV$_{BC}$ – Volume of fluorescence in Brain Capillaries (Voxels of AF680 Co-localized with FITC voxels)

TAV$_{BP}$ - Volume of fluorescence in Parenchyma (TTAV minus TTA$_{BC}$)

VTA$_{BC}$ - Volume fraction of Test Article in Brain Capillaries (TAV$_{BC}$ / TTV)

VTA$_{BP}$ - Volume fraction of Test Article in Brain Parenchyma (TAV$_{BP}$ / TTV)

VTA$_{TOT}$ – Volume fraction of Test Article in the Brain Capillaries and Parenchyma (TTAV / TTV)

Supplemental Table 2. Quantitation of stroke infarct size by TTC assay

Trial #1

Treatment	Animal #	Absorb, Ischemic	Absorb, Contralateral	Infarct Volume (% loss)
Sham	1	0.163	0.162	-0.23
	2	0.161	0.161	0.02
	Mean			-0.10
	SEM			0.12
PBS	3	0.093	0.173	46.34
	4	0.042	0.085	50.41
	5	0.122	0.218	44.04
	Mean			46.93
	SEM			1.86
NOX4 siRNA Alone	7	0.068	0.150	54.76
	8	0.068	0.121	43.39
	9	0.098	0.205	52.36
	10	0.097	0.162	39.81
	Mean			47.58
	SEM			3.56
MTfp-NOX4 siRNA	11	0.105	0.167	37.32
	12	0.072	0.111	35.14
	13	0.146	0.207	29.31
	Mean			33.92
	SEM			2.39

Trial #2

Treatment	Animal #	Absorb, Ischemic	Absorb, Contralateral	Infarct Volume (% loss)
Sham	50	2.615	2.907	10.04
	51	1.904	2.077	8.33
	53	1.788	1.581	-13.09
	Mean			1.76
	SEM			7.44
PBS	46	0.630	1.117	43.60
	47	0.578	1.260	54.13
	55	0.356	0.862	58.70
	Mean			52.14
	SEM			4.47
MTfp-scramRNA	59	0.571	1.550	63.16
	60	0.902	1.301	30.67
	65	0.577	0.956	39.64
	Mean			44.49
	SEM			9.69

Supplemental Table 3: Raw data and calculations for Figure 3. The qPCR values were compared using the method described by Huggett et al. (2005)

Raw data for Left ischemic side	Gene	Ct	ΔCt (target-reference)	Average of ΔCt	ΔΔ Ct = ΔCtsample-Average ΔCt control PBS Sham	2eΔΔCt	Average of 2eΔΔCt	SD of 2eΔΔCt	SEM of 2eΔΔCt	Ratio of Ave 2eΔΔCt (sample/sham)	Ratio of Ave 2eΔΔCt (sample/PBS stroke)
PBS-sham	Nox4	32.47	15.60	15.215	0.385	0.766	1.036	0.382	0.270	1.000	0.663
PBS-sham	Nox4	31.86	14.83		-0.385	1.306					
PBS-stroke	Nox4	30.26	14.64	14.57	-0.575	1.490	1.562	0.083	0.048	1.508	1.000
PBS-stroke	Nox4	30.68	14.59		-0.625	1.542					
PBS-stroke	Nox4	30.26	14.49		-0.725	1.653					
siRNA-stroke	Nox4	32.17	15.10	14.56	-0.115	1.083	1.617	0.370	0.185	1.561	1.035
siRNA-stroke	Nox4	32.35	14.36		-0.855	1.809					
siRNA-stroke	Nox4	32.13	14.28		-0.935	1.912					
siRNA-stroke	Nox4	32.01	14.48		-0.735	1.664					
MTfp-siRNA-st	Nox4	32.75	15.04	14.86	-0.175	1.129	1.300	0.240	0.120	1.255	0.833
MTfp-siRNA-st	Nox4	30.52	14.65		-0.565	1.479					
MTfp-siRNA-st	Nox4	31.52	15.13		-0.085	1.061					
MTfp-siRNA-st	Nox4	30.73	14.60		-0.615	1.532					
pbs-sham	B-actin	16.87									
pbs-sham	B-actin	17.03									
PBS-stroke	B-actin	15.62									
PBS-stroke	B-actin	16.09									
PBS-stroke	B-actin	15.77									
siRNA-stroke	B-actin	17.07									
siRNA-stroke	B-actin	17.99									
siRNA-stroke	B-actin	17.85									
siRNA-stroke	B-actin	17.53									
MTfp-siRNA-st	B-actin	17.71									
MTfp-siRNA-st	B-actin	15.87									
MTfp-siRNA-st	B-actin	16.39									
MTfp-siRNA-st	B-actin	16.13									

Raw data for Right Contralateral side	Gene	Ct	ΔCt (target-reference)	Average of ΔCt PBS-Sham	ΔΔ Ct = ΔCtsample-ΔCtcontrol	2eΔΔCt	Average of 2eΔΔCt	SD of 2eΔΔCt	SEM of 2eΔΔCt	Ratio of Ave 2eΔΔCt (sample/sham)	Ratio of Ave 2eΔΔCt (sample/PBS stroke)
PBS-sham	Nox4	32.47	15.30	15.385	-0.085	1.061	1.002	0.083	0.059	1.000	1.004
PBS-sham	Nox4	32.76	15.47		0.085	0.943					
PBS-stroke	Nox4	32.81	15.91	15.43	0.525	0.695	0.998	0.297	0.171	0.996	1.000
PBS-stroke	Nox4	32.91	15.37		-0.015	1.010					
PBS-stroke	Nox4	34.71	15.02		-0.365	1.288					
siRNA-stroke	Nox4	34.02	16.06	15.47	0.675	0.626	0.999	0.400	0.200	0.997	1.001
siRNA-stroke	Nox4	33.86	15.33		-0.055	1.039					
siRNA-stroke	Nox4	32.55	14.76		-0.625	1.542					
siRNA-stroke	Nox4	32.95	15.73		0.345	0.787					
MTfp-siRNA-st	Nox4	32.75	15.92	15.39	0.535	0.690	1.057	0.447	0.223	1.056	1.060
MTfp-siRNA-st	Nox4	32.4	15.31		-0.075	1.053					
MTfp-siRNA-st	Nox4	33.03	15.71		0.325	0.798					
MTfp-siRNA-st	Nox4	31.77	14.63		-0.755	1.688					
pbs-sham	B-actin	17.17									
pbs-sham	B-actin	17.29									
PBS-stroke	B-actin	16.9									
PBS-stroke	B-actin	17.54									
PBS-stroke	B-actin	19.69									
siRNA-stroke	B-actin	17.96									
siRNA-stroke	B-actin	18.53									
siRNA-stroke	B-actin	17.79									
siRNA-stroke	B-actin	17.22									
MTfp-siRNA-st	B-actin	16.83									
MTfp-siRNA-st	B-actin	17.09									
MTfp-siRNA-st	B-actin	17.32									
MTfp-siRNA-st	B-actin	17.14									

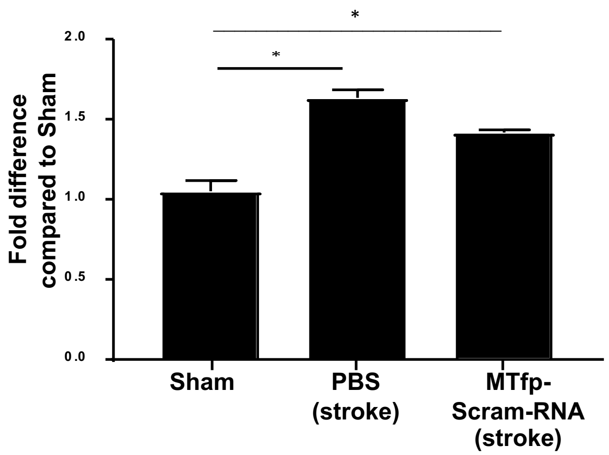

Supplemental Figure 1. NOX4 mRNA expression in the brain after MTfp-scrambled RNA treatment and stroke induction. Relative gene expression of *Nox4* in the mouse brain hemispheres by RT-PCR following treatment and 24 hours post-stroke (GAPDH as a reference gene). For each treatment group, the *Nox4* expression in the left hemisphere (stroke) is normalized against the right hemisphere (contralateral) of the same animal. Data are shown as mean ± SEM; * P-value <0.05.

Supplemental Table 4: Raw data for Supplemental Figure 2:

	Target Name	C_T
Sham Contralateral	SDHA	22.74603
	SDHA	22.73561
	GAPDH	19.22237
	GAPDH	19.26666
Sham Ischemic	SDHA	24.31267
	SDHA	24.29483
	GAPDH	20.93466
	GAPDH	20.82387
Saline Contralateral	SDHA	23.56614
	SDHA	23.58747
	GAPDH	20.34948
	GAPDH	20.4035
Saline Ischemic	SDHA	23.91395
	SDHA	23.89643
	GAPDH	20.30284
	GAPDH	20.26963
MTFp Contralateral	SDHA	24.45157
	SDHA	24.46888
	GAPDH	20.6589
	GAPDH	20.6973
MTFp Ischemic	SDHA	24.92251
	SDHA	24.9525
	GAPDH	21.22842
	GAPDH	21.28262

Average

		Contralateral	Ischemic
Sham	SDHA	22.74	24.3
	GAPDH	19.23	20.87
Saline	SDHA	23.57	23.9
	GAPDH	20.37	20.28

MTFp	SDHA	24.46	24.93
	GAPDH	20.67	21.25

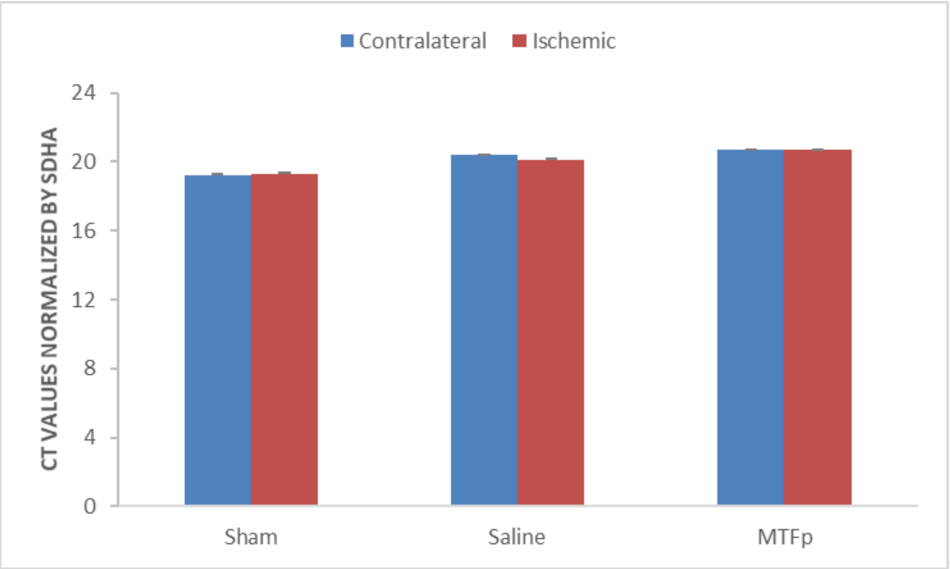

Supplemental Figure 2. GAPDH as a reliable reference gene to evaluate NOX4 mRNA expression. GAPDH expression was normalized to succinate dehydrogenase complex flavoprotein subunit A (SDHA) expression, a gene known to be a reliable reference gene in brain ischemia. Sham = surgical control (no hypoxia). Saline = surgical hypoxia induction with saline injection. MTFp = surgical hypoxia induction with MTF peptide injection. Error bars represent standard error of mean (SEM) of duplicate values. GAPDH gene expression does not significantly change after induction of ischemia in the brain, making it a reliable reference gene to evaluate NOX4 mRNA expression.

Methodology for Supplemental Figure 2:

Frozen brain tissue samples were first processed with 70 µm cell strainer to disintegrate them and immediately transferred to lysis buffer (Qiagen) and then homogenised using an 18G needles and syringes. RNA was isolated using the RNeasy® plus Mini Kit (Qiagen) in accordance with the manufacturer's instructions Subsequently RNA was quantified spectrophotometrically (NanoDrop, ThermoFisher Scientific) at 260 nm (A_{260}) and purity was estimated by an A_{260}/A_{280} ratio > 1.8. The integrity, purity, and amount of RNA were verified by visualization of total RNAs after electrophoresis on 1% agarose gel. 2 µg of RNAs were reverse transcribed using a SuperScript II Reverse Transciptase (Thermofisher Scientific) according to the manufacturer's instructions.

RT-Q-PCR reactions were carried out for GAPDH and SDHA in each sample using GeneAmp 9600 Fast Instrument (ThermoFisher Scientific). Each reaction was performed in a final volume of 20 µL containing 2µL cDNA diluted with H_2O, 10 µl of iTaq Universal SYBR® Green Supermix (Bio-Rad) and 1 µl of each gene specific primers (SDHA Fwd primer: 5'-GGAACACTCCAAAACAGACCT 3 and Rev primer" 5'-ACCACTGGGTATTGAGTAGAA-3', GAPDH Fwd primer:5'-AACTTTGGCATTGTGGAAGG-3' and Rev primer 5'-ACACATTGGGGGTAGGAACA-3'. All samples were run in triplicate and average values were calculated. Two independent reverse transcriptions were tested for each gene.
